# An incremental approach to automated protein localisation

**DOI:** 10.1186/1471-2105-9-445

**Published:** 2008-10-20

**Authors:** Marko Tscherepanow, Nickels Jensen, Franz Kummert

**Affiliations:** 1 Applied Computer Science, Faculty of Technology, Bielefeld University, Universitätsstraße 25, D-33615 Bielefeld, Germany; 2 Genetics Department, Faculty of Biology, Bielefeld University, Universitätsstraße 25, D-33615 Bielefeld, Germany

## Abstract

**Background:**

The subcellular localisation of proteins in intact living cells is an important means for gaining information about protein functions. Even dynamic processes can be captured, which can barely be predicted based on amino acid sequences. Besides increasing our knowledge about intracellular processes, this information facilitates the development of innovative therapies and new diagnostic methods. In order to perform such a localisation, the proteins under analysis are usually fused with a fluorescent protein. So, they can be observed by means of a fluorescence microscope and analysed. In recent years, several automated methods have been proposed for performing such analyses. Here, two different types of approaches can be distinguished: techniques which enable the recognition of a fixed set of protein locations and methods that identify new ones. To our knowledge, a combination of both approaches – i.e. a technique, which enables supervised learning using a known set of protein locations and is able to identify and incorporate new protein locations afterwards – has not been presented yet. Furthermore, associated problems, e.g. the recognition of cells to be analysed, have usually been neglected.

**Results:**

We introduce a novel approach to automated protein localisation in living cells. In contrast to well-known techniques, the protein localisation technique presented in this article aims at combining the two types of approaches described above: After an automatic identification of unknown protein locations, a potential user is enabled to incorporate them into the pre-trained system. An incremental neural network allows the classification of a fixed set of protein location as well as the detection, clustering and incorporation of additional patterns that occur during an experiment. Here, the proposed technique achieves promising results with respect to both tasks. In addition, the protein localisation procedure has been adapted to an existing cell recognition approach. Therefore, it is especially well-suited for high-throughput investigations where user interactions have to be avoided.

**Conclusion:**

We have shown that several aspects required for developing an automatic protein localisation technique – namely the recognition of cells, the classification of protein distribution patterns into a set of learnt protein locations, and the detection and learning of new locations – can be combined successfully. So, the proposed method constitutes a crucial step to render image-based protein localisation techniques amenable to large-scale experiments.

## Background

Although the genomes of several organisms have been sequenced, the functions of the genes' products are often unknown. The most important gene products are probably proteins. They are virtually involved in performing every kind of biological function – for instance, catalysis, cell motility and signal transduction. Furthermore, proteins are the most abundant class of macromolecules in living cells. Hence, the analysis of an organism's complete set of proteins, which is referred to as the proteome, is a crucial aim of biological sciences. One tool for achieving this goal is provided by location proteomics; i.e. the subcellular localisation of the complete set of proteins. Depending on the cell compartments that a protein occurs in, conclusions about its function can be drawn. Moreover, proteomic changes, for example those caused by the cell cycle or chemical agents, can be exploited for the diagnosis of diseases or the development of innovative therapies. Since the amino acid sequences of numerous proteins are known or can be derived from sequenced genomes, these amino acid sequences have been exploited to predict the proteins' locations. In order to accomplish this task, features such as targeting signals, which control the transportation, specific structural elements as well as information on homologous proteins are analysed. A comprehensive summary of prediction methods has been published by Chou and Shen [1].

Unfortunately, dynamic changes and differences between cell types are very difficult to predict based on a protein's amino acid sequence [2]. Therefore, it is beneficial to determine protein locations in intact, living cells. Here, even proteins with unknown targeting signals can be observed and associated with visible cell organelles.

In order to visualise the proteins, they are fused with fluorescent proteins. Afterwards they can be observed by means of a fluorescence microscope [3]. The proteins' spatial distribution leads to characteristic location patterns or rather protein distribution patterns, which correspond to the cell compartments that enclose the proteins. The tagging itself can be performed in a way which is amenable to high-throughput processing [2,4]. Furthermore, it is not necessary to know the proteins' amino acid sequences in advance. As in complex organisms, for instance mammals, the number of expressed proteins surpasses the number of genes considerably, methods allowing for the fast analysis of a large number of proteins are required. Nowadays, biologists frequently perform the subcellular localisation of proteins by hand [5]. In several experiments, the investigation is supported by auxiliary microscope images [6] or image-editing software [4]. But such manual evaluations of protein distribution patterns can barely account for the large number of existing proteins. Furthermore, they are subject to the experimenters' training and experience. Therefore, automated techniques which are amenable to high-throughput processing are required. In the literature, several aspects of such techniques have been considered:

### Classification of Protein Locations

The classification of protein distribution patterns into a set of pre-defined locations is the most common approach to determine protein locations. Here, the research has been dominated by Murphy and his group. They have experimented with a multitude of different methods – in particular, microscopy techniques [7], numerical features [8-10], feature reduction methods [11] and classifiers [7,12]. They usually consider ten different cell compartments [7-11] if two-dimensional images are applied. Using three-dimensional images, up to eleven different protein locations [7,13] have been examined. An elaborate summary of their approaches and the current progress in the field of automated location determination is given in [14].

In recent years, the interest in developing automated methods for the subcellular localisation of proteins has increased. Conrad and his colleagues proposed a feature-based machine learning approach to the analysis of twelve protein locations in single-cell images of live human cells [15]. Here, two locations (ER and microtubules) were recognised with an accuracy of less than 50%. As an alternative to using numerical features, Danckaert and her colleagues suggested the usage of a neural network structure, which enables the recognition of six protein locations using down-scaled images of single cells [16].

Other researchers have employed techniques tailored to specific locations: Kasson and his co-researchers proposed a technique for the classification of proteins localised at the plasma membrane [17]. Schiffmann and his colleagues used counterstaining in order to measure the protein concentration in the kinetochores (cell compartments involved in mitosis) [18]. A similar method has recently been applied by Raman and his co-researchers [19]. They have analysed abnormalities of nuclear compartments called centrosomes by means of counterstaining the nucleus and exploiting radial symmetries. Liebel suggested a simple image-processing-based technique enabling large-scale screens of proteins localised in the Golgi apparatus [20]. Logg and her colleagues investigated proteins located in the nucleus [21].

In contrast to subcellular location prediction based on amino acid sequences (cf. [22]), the classification of observed protein distribution patterns does not require a differentiation between proteins simultaneously occurring in a single location or at multiple locations. Rather, appropriate classes representing double locations have to be defined. Furthermore, moving proteins can be captured by observing the protein distribution over time and repeating the classification.

### Identification and Grouping of New Locations

In addition to assigning protein distribution patterns to a fixed set of locations, the interest in automatically identifying and grouping distinct location patterns has risen. It has been particularly pushed forward by Murphy's group [2,8,23-25]. Here, the applied techniques differ considerably from the ones used for protein localisation so far: Supervised learning mechanisms have been replaced by unsupervised ones, which do not incorporate prior knowledge about the data processed. These unsupervised learning methods summarise similar location patterns into so-called clusters. In comparison to classifiers, the number of clusters typically exceeds the number of classes or rather the number of actual protein locations. In principle, the whole process is controlled by a similarity criterion, which reflects the similarity of the regarded images in the feature space and determines the outcome.

In order to find relevant location patterns, Murphy's group proposed a method, which distinguishes between all proteins under analysis, even if they share a common location [2,23]. They use the k-means algorithm to cluster the images independently of the proteins shown. Then all images showing a specific protein are analysed. A protein is associated with the cluster containing the majority (minimum 33.3%) of the corresponding images. All other images (up to 66.6%) are dropped. If no cluster comprises more than 33.3% of a protein's images, the respective location patterns are discarded completely. So, stable connections between created clusters and proteins are established. In addition to the k-means algorithm, they apply hierarchical clustering to the location patterns that have not been dropped before. Here, it is assumed that the clusters yielded by the systems with the highest agreement reflect the real structure of the data. Information on known protein locations is not incorporated.

### Recognition of the Surrounding Cells

The analysis of tagged proteins in live cells is a difficult task. A fluorescence image typically contains multiple or even numerous cells. These cells may be in various states resulting in different locations of the proteins. Furthermore, the cells themselves are not necessarily visible. Thus, a corresponding fluorescence micrograph contains bright spots corresponding to accumulations of tagged proteins. These spots vary in size and shape. But they cannot be associated with specific cells and locations therein. A trained biologist might be able to estimate the position of the surrounding cells. However, in an automatic context, additional information is necessitated. This knowledge is usually acquired by considering additional images, e.g. fluorescence images of stained nuclei, cell membranes or cytoplasms [14]. Unfortunately, if such dyes are utilised with living cells, they may interfere with examined proteins or even kill the cells. Furthermore, additional fluorescence channels are occupied. In order to circumvent these drawbacks, we have developed a cell recognition approach based on bright-field images [26-28]. Provided that a cell has been recognised in such a bright-field image, the corresponding image region of the fluorescence micrograph can be examined concerning protein distribution patterns. A similar approach has recently been proposed by Logg and her colleagues and exemplified for two proteins located in the nucleus [21].

### Our Approach

A combination of exploiting available biological knowledge and enabling the incorporation of new information would be more suitable with respect to large-scale analyses than the approaches discussed above. In such large-scale experiments unknown locations are likely to show up and must be processed. Otherwise, they result in errors. Therefore, in this article, a technique is introduced, which does not distinguish between the classification of known protein locations and the identification and grouping of new ones; for example, if it is trained to recognise proteins situated in the lysosomes, it will reject images of proteins located in the mitochondria as unknown. These unknown distribution patterns can be grouped according to their similarities or labelled by an expert and incorporated into the running system. In order to ensure that the created classes have a biological meaning, we favour the approach based on manual labelling. Therefore, we do not have to drop a large fraction of the available data as proposed by Murphy's group [2,23]. In addition, such techniques which are solely based on the usage of a similarity criterion in the feature space cannot guarantee the biological significance.

Besides fusing two different approaches to protein localisation, the proposed protein localisation technique has been adapted to the automated cell recognition method we have published in [26-28]. So, a completely automatic processing of microscope images becomes possible: The cells of interest are found, known protein locations are recognised and unknown locations are sorted out for a further analysis. However, the labelling of unknown protein distribution patterns has to be performed by experts. But this labelling does not need to take place during the application of our protein localisation method. Rather, all unknown images, which have been sorted out, are collected and can be examined off-line after an experiment has finished.

## Methods

### The Applied Cell Line

The cells, the microscope images of which are analysed within the scope of this paper, stem from the fall army worm *Spodoptera frugiperda *– a moth inhabiting the northern hemisphere. In 1977 a cell line called IPLB-SF-21 was extracted from immature ovaries of *Spodoptera frugiperda *pupae [29]. It served as a basis for the derivation of the utilised cell line termed Sf9 [30].

Insect cells have been proven to be beneficial for the high-level expression of foreign proteins [31-33]. Here, the proteins are often correctly modified and localised. Genetic engineering enables the proper processing of additional proteins [34]. Besides their application to protein expression, insect cells have been studied with respect to insect pest management [33]. As a result, a large number of cell lines originating from several insect species are available [29,31].

Sf9 cells have several beneficial features, which make them amenable to high-throughput investigations. First of all, they are robust and not very demanding; for example, they grow at room temperature and in serum-free medium without any added growth factors [35]. In addition, Sf9 cells exhibit a round shape with diameters between about 15 *μ*m and 20 *μ*m, which is relatively large. Cells from the budding yeast *Saccharomyces cerevisiae*, for example, reach only diameters of about 8 *μ*m [36]. So the differentiation between protein localisation patterns is alleviated if Sf9 cells are employed. In contrast to mammalian cells, the cell growth is independent from carbon dioxide. As a result, no special devices are required. Finally, they are adherent, i.e. they form a single layer attached to a surface. Through this, the application of automatic techniques such as auto-focus or image analysis procedures is facilitated.

As known protein localisation approaches have been applied to alternative cell types (e.g., HeLa [16] or 3T3 cells [2]), the methods used for analysing the protein distribution patterns had to be modified. But the proposed usage of Sf9 cells alleviates the automatic analysis considerably.

### Image Acquisition

Since fusion proteins obtained using fluorescent proteins such as green fluorescent proteins (GFPs) and yellow fluorescent proteins (YFPs) were only available for a subset of the considered locations, conventional organelle probes had to be applied so as to simulate further locations. Otherwise, a reasonable variety of protein distribution patterns could not have been collected. But as the resulting fluorescence micrographs are assumed to be very similar to the corresponding protein distribution patterns, the negative influence of this procedure is limited. Table 1 lists the protein locations and the respective dyes.

**Table 1:** Applied staining methods.

**cell compartment**	**dye**
cytoplasm including nucleus	GFP without fusion
cytoplasm without nucleus	GFP fused to a cytoplasmic protein
endoplasmic reticulum (ER)	200 nM DiOC_5_(3)
lysosomes	2 *μ*M LysoSensor™ Green DND-153
microtubules	1 *μ*M Oregon Green Paclitaxel
mitochondria	1 *μ*M MitoTracker^® ^Orange CM-H_2_TMRos
nucleoli	YFP-Nop56
nucleus	3 *μ*M Hoechst 33342
peroxisomes	GFP-SKL
plasma membrane	6 *μ*M FM 1–43

The images depicting several protein localisations were obtained by constructing fusion proteins, while others were acquired using conventional dyes.

All organelle stains were obtained from Molecular Probes. Before staining or transfection, the cells were plated in 24-well glass-bottom plates. For all stains except for the plasma membrane stain the cells were incubated for 30 minutes with the appropriate dye concentration and washed three times with medium. For the FM dye the images were taken 5–15 minutes after the addition of the dye without washing steps. In order to visualise the cytoplasm including the nucleus, a GFP was used directly, without creating a fusion protein. The cytoplasm excluding the nucleus was depicted by means of expressing a GFP fused to cytoplasmic proteins. The peroxisomes were marked by a GFP with a peroxisomal targeting sequence consisting of the carboxy-terminal-fused amino acids serine, lysine and leucine [37]. The resulting *GFP-SKL *as well as the *GFP*s themselves were amplified from pEGFP-Tub (Clontech) with the primers GGATCCATGGTGAGCAAGGGCGA, CTCGAGTTACTTGTACAGCTCGTCCATGC and CTCGAGTTAAAGCTTGCTCTTGTACAGCTCGTCCATGC. After amplification the constructs were cloned into pGEM-T Easy. The genes were then cut by *Nco*1 and *Xho*1 and ligated into the insect expression vector pIEx4 (Novagen). The nucleoli were visualised with a YFP fusion to the *Medicago truncatula *homologue of the Nop56 protein. Nop56 is a nucleolar protein required for ribosomal subunit synthesis [38]. It was amplified from *Medicago truncatula *RNA by RT-PCR with the primers ATTCTCGAGTAATGGCACTCTTTCTCCC and ACTAGGATCCTTATTCAGCATCCTTCTTTT and cloned via *Xho*1 and *Bam*H1 in frame with the *YFP *open reading frame into pEYFP-C1 (Clonetech). The fusion construct was then cut with *Nco*1 and *Bam*H1 and ligated into pIEx4.

The Sf9 cells were obtained from Novagen and maintained in serum-free BacVector^® ^Insect Cell Medium at 25°C. Their transfection was performed using the constructed expression vectors and Insect Gene Juice (Novagen) in 24-well glass-bottom plates according to the manufacturer's protocol. The fluorescence patterns were visualised directly after staining or 36 hours after transfection with an Olympus IX81 microscope equipped with appropriate filters. In addition to the fluorescence images showing protein distribution patterns, bright-field images were taken in parallel. These bright-field images can be applied to find the surrounding cells. All images were captured with a 60 × objective (numerical aperture: 0.9) by the scan^R ^Software (Olympus Soft Imaging Solutions GmbH).

### Representation of Protein Distribution Patterns

Observed protein distribution patterns can be represented in several ways. In comparison to the direct application of pixel intensities, the usage of numerical features has proven particularly advantageous for the classification of fluorescence images showing tagged proteins [8,10]. From the literature, a multitude of different feature sets is known [9,10,15]: morphological features, histogram-based features, edge-related features, convex hull features, moment-based features, features based on co-occurrence matrices and wavelet features. Here, usually several types of features are required to allow for a correct classification of protein location patterns. So, if a new cell line, such as Sf9, is to be analysed, the problem consists in defining a set of feature types which reflects the location patterns adequately. Selecting only an individual feature type most probably does not surface. Since from the resulting set several features may be irrelevant or redundant, a feature reduction step has been shown to be beneficial [14]. Here, the application of feature reduction methods requires the availability of appropriate datasets. These datasets are further necessary for the classification of the protein distribution patterns; that is, their association with a distinct set of protein locations.

#### Features

In the literature, subcellular location features are either computed from images of single cells or multi-cell images. Single-cell images usually have been cropped manually [9,13,39] or determined automatically using counterstaining [9,13]. In contrast, multi-cell images [40] are evaluated as a whole and do not rely on any kind of segmentation or cropping. But they require homogeneous location patterns for all cells, which cannot be guaranteed for numerous biological experiments. Furthermore, image properties different from protein locations, e.g. the cell distribution, might influence the outcome of the analyses. Therefore, we propose the usage of a mask image, called a cell mask, for each cell. These cell masks depict all pixels belonging to a specific cell in white and all other pixels in black. So, the protein distribution patterns of all cells can be analysed individually, even if the cells lie in cell clusters. But since only pixels within the considered cells are regarded, feature sets that are based on rectangular image regions cannot be transferred easily.

**Figure 1:**
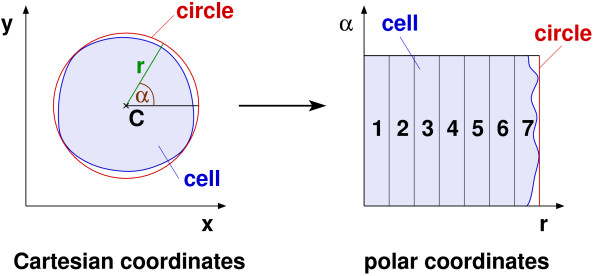
**Considered image regions**. Each cell, defined by its cell mask, is mapped to a circle with the centre *C *and transformed into polar coordinates (r, *α*). Then, seven regions of equal width are analysed. In a Cartesian coordinate system, these regions correspond to disjoint rings.

Since the applied cell line as well as the proposed mask images have not been applied within the frame of an automated protein localisation technique before, the known feature sets are only of limited use, although individual features are transferable. Consequently, we have chosen an own feature set. We have selected the corresponding features in such a way as to enable a comprehensive description of all protein distribution patterns which might appear in Sf9 cells: Firstly, features enabling a consideration of the positions of tagged proteins relative to the surrounding cells are employed. They comprise the well-known Zernike moments [41] and region-dependent texture features, which were developed by us. While Zernike moments quantitate the positions of bright image structures by means of complex radial polynomials, the region-dependent texture features employ simple grey-level statistics to ring-shaped image regions: the mean, the variance, the skewness, the uniformity, the smoothness, the entropy and the median. Using polar coordinates, the rings can be determined easily (see Figure 1).

Secondly, morphological features are employed, in particular pattern spectra [42], which allow for an evaluation of the shape and the size of protein accumulations. In contrast to alternative morphological features, e.g. the features proposed in [8] and [40], a prior binarisation or segmentation of intracellular structures is not required. Pattern spectra are particularly useful for the description of small nearly circular cell organelles such as lysosomes or peroxisomes.

Finally, general properties of the protein distributions are examined by means of fractal features [43] and histogram-based statistical features which are applied to the whole cell. So, characteristics less obvious than shape, size and location of protein accumulations are incorporated into the localisation procedure, for example, the heterogeneity and roughness of the image at different scales.

All of these features have been applied in such a way that an incorporation of the respective cell mask is enabled. Using Zernike moments and region-dependent texture features, each cell is mapped to a circle, which resembles the shape of the Sf9 cells. Using the other types of features, pixels not contained in the respective mask are explicitly neglected.

#### Basic Datasets

**Figure 2:**
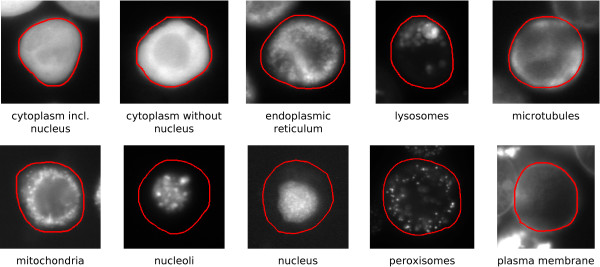
**The ten protein locations considered**. The red contours represent the surrounding cells, which were manually extracted from corresponding bright-field images by a biological expert. Several distribution patterns, e.g. for the endoplasmic reticulum and the microtubules, resemble each other very closely.

As a basis for all further investigations, basic datasets containing feature vectors of images showing ten major protein locations were generated. These locations correspond to specific cell compartments or combinations thereof. Exemplary micrographs are shown in Figure 2. The respective numbers of cell masks, which had been manually extracted from corresponding bright-field images by a biological expert, are summarised in Table 2. These cell masks were associated with protein distribution patterns from corresponding fluorescence micrographs. As the number of feature vectors equals the number of cell masks, a total of 1, 326 samples were available.

**Table 2:** Numbers of cell masks for the regarded protein locations.

**cell compartment**	**cell masks**
cytoplasm including nucleus	144
cytoplasm without nucleus	56
endoplasmic reticulum (ER)	142
lysosomes	222
microtubules	102
mitochondria	268
nucleoli	74
nucleus	150
peroxisomes	71
plasma membrane	97

For each cell compartment a certain number of cell masks was extracted from bright-field images. But in order to realise a protein localisation, an analysis of fluorescence micrographs must be performed. Therefore, each cell mask has been associated with a corresponding region in a fluorescence micrograph that depicts a protein distribution pattern characteristic for this compartment.

In order to characterise the protein distribution patterns, we compiled two different feature sets. They are referred to as feature set A and feature set ℬ, respectively. Using each feature set, an individual basic dataset was created. Both feature sets comprise pattern spectra, fractal features and histogram-based features. In addition, feature set A encompasses Zernike moments and feature set ℬ region-dependent texture features resulting in a total of 73 basic features each. As the size of both feature sets is equal, a comparison of the established but computationally intensive Zernike moments [9,44] with our simpler region-dependent texture features becomes possible. Using an AMD Athlon™ 64 processor (2 GHz, 32-bit mode), the mean time for computing feature set A and ℬ for one of the 1, 326 cell masks amounts to 4.39 s and 2.14 s, respectively. So, the suggested region-dependent texture features are a promising alternative to Zernike moments.

#### Automatic Data Generation

The 1, 326 manually extracted cell masks may differ from cell masks, which are automatically determined using a cell recognition approach. In particular, the cell boundaries are likely to vary slightly, which might influence the outcome of the protein localisation. But in an automated context, proteins have to be localised in such automatically acquired cell masks. In order to analyse and solve this problem, we propose the application of a procedure enabling the automatic generation of additional training data. It was successfully applied within the scope of a cell recognition approach before (see [26,27]) and works as follows: The cells which are described by manually determined cell masks are automatically segmented in the respective bright-field images. The resulting segments are associated with these cell masks; i.e., the segments constitute variations of the masks. Provided that the difference between a segment's contour and the respective cell mask's contour is less than 10% of the manually segmented cell's diameter, the automatically determined image region is accepted as an additional cell mask. If the resulting, automatically determined cell masks are superimposed onto the corresponding fluorescence micrographs, variations of the original protein distribution patterns can be acquired. So we generated an additional 12, 015 samples. These segments are more likely to occur than the manually determined cell masks if the proposed protein localisation technique is applied in conjunction with the considered cell recognition procedure. As a result, the automatic generation of training data alleviates the cooperation of both procedures. In addition, the number of samples was increased, which facilitates the classification task. Otherwise, the number of training samples might not have been sufficient.

#### Feature Reduction

Suitable features were selected by means of the stepwise discriminant analysis (SDA). It chooses a set of features depending on statistical properties of the data. The used classifier is not taken into account. However, from the literature it is known that the stepwise discriminant analysis is very well-suited for selecting features in the context of protein localisation [11,15]. In order to achieve comparable results, the procedure STEPDISC of the software package SAS/STAT [45] was applied here.

### Classification of Known Locations

The actual protein localisation is performed by classifying observed protein distribution patterns in classes corresponding to protein locations; that is, observed protein distribution patterns are assigned to one of the ten locations depicted in Figure 2. Here, we propose the application of an extended version of the simplified fuzzy ARTMAP (SFAM) originally introduced in [46] as a classifier. The SFAM has several advantages in comparison to alternative classifiers such as multilayer perceptrons [7,12,15,47] and support vector machines [7,15,44,47], which are usually applied within the scope of protein localisation: It is very well-suited to fast and stable on-line learning, and enables the detection, clustering and incremental learning of unknown samples, which is crucial with respect to the desired ability of incorporating new protein location patterns into the trained system. Moreover, it is directly applicable to multi-class classification problems. Eventually, the SFAM's classification accuracy is comparable to other state-of-the-art methods [26,48].

**Figure 3:**
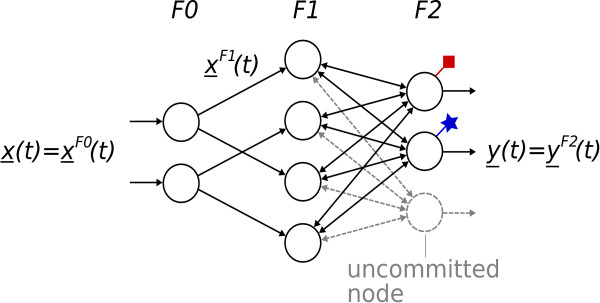
**Structure of the SFAM**. The SFAM encompasses three layers: *F*0, *F*1 and *F*2. The neurons of the *F*2 layer are associated with class labels. Furthermore, there are uncommitted nodes, which can be incorporated if new input vectors are to be learnt.

The SFAM has a three-layered architecture (see Figure 3). The first layer *F*0 performs an encoding of the input vector *x*(*t*) called complement coding. The resulting vector *x*^*F*1^(*t*) constitutes the input vector of the subsequent layer *F*1. The nodes of the output layer *F*2 are linked to all nodes of the *F*1 layer. The corresponding weights ${\underline{w}}_{i}^{F2}(t)$ define hyper-rectangular subspaces of the input space – the categories. The categories' sizes are limited by means of the vigilance parameter *ρ*. Moreover, each *F*2 neuron is associated with a class label. As the SFAM is an incremental network, there are neurons, which are not in use but required for an extension of the network – the uncommitted nodes. During the training process, they are incorporated if the available nodes are not able to represent an input vector.

In order to classify a presented feature vector, all *m *outputs are initialised by -1 first. Afterwards, the activation of all *F*2 nodes is computed and the output of the best-matching node $y_{j}^{F2}$(*t*) is set to its class label. The other nodes remain unchanged. Thus, a classification result *c*(*t*) can be determined by:

(1) $$c(t)=\underset{i=1,...,m}{\max}y_{i}^{F2}(t).$$

As the presented approach is not only intended to localise a fixed set of proteins, a measure specifying whether an input vector is known or unknown is required. So, previously unknown locations can be recognised and incorporated into the system. Furthermore, it would be beneficial to have information on the degree of knowledge so as to find related or similar protein locations. Unfortunately, the original activation $z_{i}^{F2}(t)$ proposed by Carpenter [49] cannot fulfil this task, since it varies depending on the size of a category *i *represented by ${\left|{\underline{w}}_{i}^{F2}(t)\right|}_{1}$.

(2) $$z_{i}^{F2}(t)=\frac{{\left|{\underline{x}}^{F1}(t)\wedge {\underline{w}}_{i}^{F2}(t)\right|}_{1}}{\alpha +{\left|{\underline{w}}_{i}^{F2}(t)\right|}_{1}}$$

Here, |·|_1 _denotes the city block norm and and ∧ symbolises an element-wise minimum operation. The choice parameter *α *is usually set slightly higher than zero, which increases the influence of the category size even more: For *α*> 0, small categories are preferred to large ones.

In order to determine an activation of the *F*2 neurons, which is independent of the categories' sizes, we decided to employ the alternative measure ${\tilde{z}}_{i}^{F2}(t)$ if an input vector is to be classified [27]. ${\tilde{z}}_{i}^{F2}(t)$ corresponds to the distance from an input vector to category *i *according to the city block norm.

(3) $${\tilde{z}}_{i}^{F2}(t)={\left|\left({\underline{x}}^{F1}(t)\wedge {\underline{w}}_{i}^{F2}(t))-{\underline{w}}_{i}^{F2}(t\right)\right|}_{1}$$

**Figure 4:**
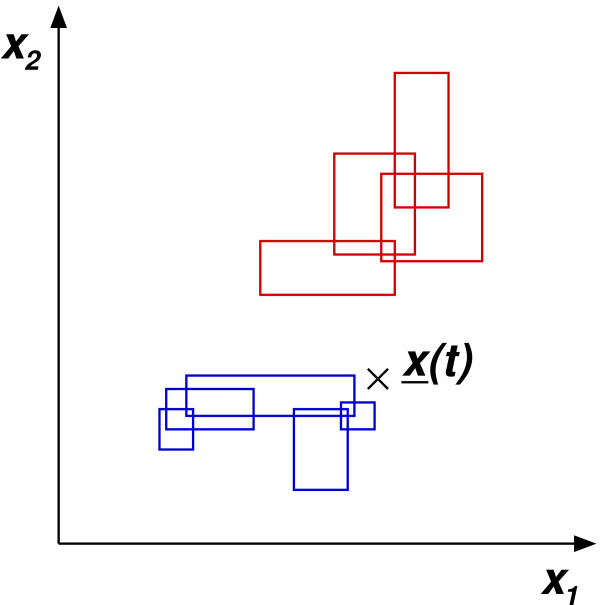
**Classification using the modified SFAM**. A new input vector *x*(*t*) is presented to an SFAM network which performs a separation of two classes in a two-dimensional feature space. The categories belonging to each of the classes are depicted in blue and red, respectively. In principle, *x*(*t*) would be unknown to the network, as it does not lie inside any category. However, the consideration of ${\tilde{z}}_{\min}^{F2}(t)$ enables the input vector to be assigned to the blue class if its distance to the next blue category is smaller than the threshold *τ*.

The minimum value ${\tilde{z}}_{\min}^{F2}(t)$ of ${\tilde{z}}_{i}^{F2}(t)$ over all *F*2 nodes *i *indicates the degree of knowledge about an input vector. Assuming ${\tilde{z}}_{\min}^{F2}(t)$ = 0, the input vector lies within a category; i.e., it is known completely. Higher values correspond to less knowledge. However, an input vector which is close to a category is likely to be representable by it. Therefore, we introduced a threshold *τ*, which denotes the maximum distance up to which an input vector is considered as being known (see Figure 4). In the case that ${\tilde{z}}_{\min}^{F2}(t)$ is larger than *τ*, the output $y_{j}^{F2}$ of the best-matching neuron remains unchanged after the initialisation. As a result, *c*(*t*) yields a class label for known input vectors and -1 otherwise (cf. Equation 1).

If *τ *equals zero, our extended SFAM is identical to the original SFAM. In principle, *τ *constitutes an additional degree of freedom, which is to be optimised in conjunction with the vigilance parameter *ρ *in order to reach high classification accuracies. But in addition, *τ *is essential to parametrise the rejection of unknown inputs.

### Detection and Incorporation of New Locations

In principle, the SFAM is able to incorporate new data (from known as well as hitherto unknown classes). Provided that the distribution patterns are presented in random order, there is no decrease in accuracy. But as the proposed approach aims at creating a method which is amenable to high-throughput processing, user interactions cannot be performed when a new input occurs; only post-processing would be possible. Thus, unknown protein distribution patterns have to be sorted out for a future inspection by an expert. Without such a manually assigned class label, these patterns might not have any biological meaning. Dissimilarity in the feature space is a necessary but not sufficient criterion to create new classes, since it depends on the employed features and similarity measures rather than biological knowledge. Furthermore, a manual association of clusters with specific protein locations would necessarily be biased by the applied protein localisation technique and the expectations of its users. Only pre-labelled data, which are used to analyse the proposed technique, ensure an objective evaluation.

Using the SFAM, an unknown location is characterised by a minimum activation ${\tilde{z}}_{\min}^{F2}(t)$ higher than a threshold *τ*. The corresponding feature vector has a distance larger than *τ *to the closest category. However, if *τ *is chosen in such a way as to maximise the classification accuracy, the vast majority of fluorescence images showing the protein locations used for training are considered as known and classified accordingly. So, if a new location resembles a known one, the corresponding feature vectors are likely to be regarded as known and classified incorrectly. In order to circumvent this problem, we introduced a second threshold *τ*_2 _with respect to ${\tilde{z}}_{\min}^{F2}(t)$. It is smaller than *τ *and defines the minimum distance to the closest category that a pattern must have in order to be recognised as potentially unknown, or rather as a possibly new protein location. In this case, an expert could be asked for advice.

*τ*_2 _must be selected in such a way that a compromise between correctly classifying the known locations and detecting potentially unknown locations is reached. In particular, the amount of required manual assignments should be minimal, although the user interventions occur after the actual experiment. With respect to the unknown locations, the fraction of inputs recognised as potentially unknown (*f*^u^) should be high. So, the new locations can be detected. On the other hand, images of already known classes should not be regarded as potentially unknown. The corresponding results are symbolised by *f*^k^. Due to these considerations, an optimal value $\tau_{2}^{\text{opt}}$ for *τ*_2 _can be determined by maximising the difference between *f*^u ^and *f*^k^:

(4) $$\tau_{2}^{\text{opt}}=\underset{\tau_{2}}{\max}(f^{\text{u}}-f^{\text{k}})$$

In addition, *τ *is still usable for the classification. So, even if ${\tilde{z}}_{\min}^{F2}(t)$ for a specific feature vector is higher than *τ*_2_, a suggestion for a likely protein location can be made unless ${\tilde{z}}_{\min}^{F2}(t)$ is higher than *τ *as well.

### Evaluation Methods

This section introduces all of the methods that were applied so as to evaluate our approach. Firstly, several accuracy measures are discussed, concerning the quantitative evaluation. Afterwards, we describe the methods for analysing our protein localisation approach with respect to both tasks which we aim to solve: the classification into a pre-trained set of locations as well as the identification and incorporation of new protein locations.

#### Accuracy

In order to contrast the classification results, the total accuracy ACC, is utilised. It has been applied by numerous researchers to this task before and constitutes a standard measure (cf. [16,17,22,44]). The total accuracy reflects the amount of correctly classified patterns. In terms of location-specific accuracies ACC_*i*_, it can be written as follows:

(5) $$\text{ACC}=\frac{1}{N}\sum_{i=1}^{n}N_{i}{\text{ACC}}_{i}$$

Here, *N *denotes the total number of available test patterns, *n *the number of regarded locations and *N*_*i *_the number of images showing location *i*. Equation 5 illustrates that ACC has a crucial drawback regarding the evaluation of classifiers: Insufficient results with respect to some protein locations might be balanced by others. So especially locations for which only a few training samples are available (i.e., *N*_*i *_is small) can be incorrectly classified without a significant impact on ACC. This is impressively shown in [44] where a total accuracy of 81% is reported but from 5 of 20 locations not even one image was recognised and for 5 other locations only results below 50% were reached. Here, the arithmetic mean ${\bar{\text{ACC}}}_{\text{am}}$ of the location-specific accuracies, which amounts to 43.1%, reflects the classification accuracy more appropriately.

(6) $${\bar{\text{ACC}}}_{\text{am}}=\frac{1}{n}\sum_{i=1}^{n}{\text{ACC}}_{i}$$

However, ${\bar{\text{ACC}}}_{\text{am}}$, which has been applied as an alternative to ACC [7,10], does not completely solve the balancing problem. Assuming that all distribution patterns of 9 out of 10 locations would be correctly recognised (ACC_*i *= 1...9 _= 100%), the minimum of ${\bar{\text{ACC}}}_{\text{am}}$ equals 90%, even if the images showing the tenth location were misclassified completely (ACC_*i *= 10 _= 0%). So, if the arithmetic mean was used for parameter optimisation, for example for the parameters of the employed classifier, an equally correct recognition of all locations could not be guaranteed. In order to circumvent this problem, here we propose to employ the mean accuracy ${\bar{\text{ACC}}}_{\text{hm}}$ in addition to the total accuracy. It denotes the harmonic mean of the location-specific accuracies ACC_*i*_.

(7) $${\bar{\text{ACC}}}_{\text{hm}}=\frac{n}{\sum_{i=1}^{n}\frac{1}{{\text{ACC}}_{i}}}$$

The harmonic mean is always smaller than or equal to the arithmetic mean: The larger the difference between individual accuracies ACC_*i*_, the more it decreases in comparison to the arithmetic mean. In principle, other measures, such as the geometric mean, would have been possible as well. But, we decided to use the harmonic mean, as it punishes unbalanced results more strongly. This is particularly important, since we perform the optimisation of the SFAM's parameters based on ${\bar{\text{ACC}}}_{\text{hm}}$ in order to ensure that the differences in accuracy between all locations are as small as possible. In comparison to using the arithmetic mean, the resulting classifiers' performances are easier to assess, as the given overall accuracies (ACC and ${\bar{\text{ACC}}}_{\text{hm}}$) reflect the location-specific accuracies ACC_*i *_more precisely. In order to simplify the mathematical notation, ${\bar{\text{ACC}}}_{\text{hm}}$ is written $\bar{\text{ACC}}$ in the following.

#### Evaluation of the Classification Task

The training was performed without distinguishing between manually and automatically acquired protein distribution patterns. But the results concerning both types of sample were computed independently, so as to enable an evaluation of the cooperation of our protein localisation technique with the applied cell recognition method.

In order to assess the proposed protein localisation approach with respect to the classification of the protein distribution patterns in the ten regarded locations, the basic datasets were randomly mixed and split into ten subsets. No dataset contains masks of cells which are part of another dataset. Using these subsets, five groups of eight training and two test datasets each were created. Here, the selection of the test datasets occurred in such a way that they were disjoint. So, it became possible to apply eight-fold cross-validation to the optimisation of the classifiers' parameters (vigilance parameter *ρ *and threshold *τ*) and five-fold cross-validation to test the localisation results.

#### Evaluation of the Retraining Task

According to the evaluation of the classification task, manually and automatically determined protein distribution patterns were not distinguished with respect to the classifiers' retraining. But the evaluations concerning the quality of the protein localisation were carried out considering only the manually acquired samples to simplify the following discussions.

In order to simulate the occurrence of protein distribution patterns showing new locations during a biological experiment, we partitioned the available data according to the respective cell compartments: Eight protein locations were considered as known and utilised for training a classifier as explained in the previous section. The remaining two locations served as unknown locations for the evaluation. In principle, the application of a single unknown cell compartment would have been possible as well. But the employed method is more realistic, since more than one single untrained location can be expected to exist. In total, there are 45 different possibilities of dividing ten classes into two groups of eight and two classes, respectively. They are denoted by *C*_*i*_.

**Figure 5:**
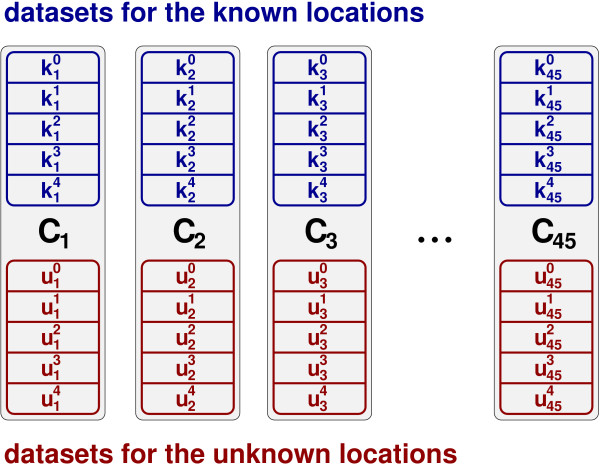
**Employed datasets**. For each combination *C*_*i *_of known and unknown cell compartments ten datasets were created. They enable five-fold cross-validation with respect to each type of protein location – known ($k_{i}^{j}$) and unknown ($u_{i}^{j}$).

The datasets of each combination *C*_*i *_of known and unknown locations were divided into five subsets (see Figure 5) so as to enable an evaluation and parameter optimisation using cross-validation. The individual datasets are referred to as $k_{i}^{j}$ and $u_{i}^{j}$ respectively. This partitioning resulted in ten datasets regarding each combination and enables cross-validation concerning the known as well as the new cell compartments.

The training of the networks with respect to the known cell compartments was performed using four of the five available datasets associated with a specific combination *C*_*i*_: Three datasets were used for training and the fourth one for validation. By iterating *ρ *and *τ*, a parameter optimisation was performed. Here, the respective fifth dataset served as an independent test set for the chosen parameter settings. This procedure was repeated for all possible groups of four datasets. On the whole, it is a simplification of the cross-validation scheme introduced in the previous section.

After finishing the training procedure, the networks' abilities to recognise unknown protein locations were evaluated. Here, the threshold *τ*_2 _was iterated from 0 to *τ *and the samples contained in $u_{i}^{0}$ to $u_{i}^{4}$ were presented to the SFAMs. Then, the fractions of unknown samples from the known (*f*^*k*^) and the new unknown protein locations (*f*^*u*^) were measured.

Once unknown protein locations have been detected, they can be incorporated in the SFAM. But, since the proposed approach is intended to be applied as a high-throughput system, no user interactions are possible during the protein localisation. However, samples recognised as an unknown location could be automatically sorted out for further assessment. After they have been associated with a protein location by a biological expert, the SFAM can be retrained. The evaluation of the retraining capabilities was performed similar to the analysis of the classification accuracies with respect to the eight previously known protein locations; i.e., four of five datasets were utilised. The remaining one was used as a test dataset. An optimisation of *ρ *and *τ *had not been necessary here, as their values were already known. The whole process was repeated for every test dataset possible. So, it constitutes five-fold cross-validation.

Besides the features themselves, the size of the feature set might affect the retraining process. Therefore, experiments based on reduced feature sets were conducted as well. However, it is virtually impossible to select a set of features, which accounts for a number of classes that are not known in advance. Therefore, this kind of feature reduction would only be of limited use with respect to the detection and learning of new cell compartments.

## Results

### Classification Task

Table 3 contrasts the localisation accuracies for systems using both feature sets with and without feature reduction by means of the SDA. The accuracies of the systems not employing feature reduction could be obtained directly. In contrast, the SDA had to be parametrised. In particular, an appropriate stopping criterion had to be chosen. Therefore, the maximum number of steps was iterated between 1 and 146, which equals twice the size of the feature sets. So it was guaranteed that any feature subset required could have been chosen. Afterwards, the number of steps that had resulted in the highest mean accuracy $\bar{\text{ACC}}$ regarding the manually extracted cells was selected. As a result, similar recognition results with respect to all regarded protein locations were ensured. In addition to $\bar{\text{ACC}}$, the number of applied features and the corresponding total accuracy ACC are given. Here, the results concerning the manually and the automatically acquired samples are distinguished.

**Table 3:** Localisation accuracies for the fixed set of protein locations.

			**I**		**II**	
				
**feature set**	**reduction method**	**#features**	**$\bar{ACC}$**	**ACC**	**$\bar{ACC}$**	**ACC**
A	-	73	0.777	0.799	0.790	0.828
ℬ	-	73	0.818	0.833	0.806	0.835
A	SDA	17	0.860	0.873	0.873	0.887
ℬ	SDA	15	0.878	0.891	0.883	0.892

Although the SFAMs were trained using both manually and automatically obtained protein distribution patterns, the evaluation is performed separately. Here, I denotes the accuracies regarding the manually acquired patterns and II the results with respect to the automatically generated samples. The results indicate an advantage of feature set ℬ in comparison to feature set A. Moreover, the feature reduction leads to a significant improvement of the protein localisation, independent from the feature set applied.

A comparison of the accuracies regarding the manually and automatically obtained samples reveals that our protein localisation technique can be successfully applied based on cell masks yielded by the automatic cell recognition approach. The results regarding the automatically generated patterns are even slightly better, which might be a consequence of their noticeably higher number.

The feature reduction led to a significant improvement of the classification results. Feature set ℬ, which employs the region-dependent texture features, seems to be more appropriate than feature set A, which is based on Zernike moments. Since the computation of feature set ℬ is significantly faster as well, it should be preferred.

In order to demonstrate the classifiers' abilities to discriminate between the ten chosen protein locations, Table 4 shows the confusion matrix of the system using feature set ℬ and feature reduction by SDA.

**Table 4:** Confusion matrix of the best system (feature set ℬ, SDA).

		**classification results (in percent)**										
		
**cell compartment**		**(a)**	**(b)**	**(c)**	**(d)**	**(e)**	**(f)**	**(g)**	**(h)**	**(i)**	**(j)**	(*)
cytoplasm + nucl.	**(a)**	**97.2**	2.1	0.0	0.7	0.0	0.0	0.0	0.0	0.0	0.0	0.0
cytoplasm – nucl.	**(b)**	17.9	**75.0**	1.8	0.0	3.6	0.0	0.0	0.0	0.0	1.8	0.0
ER	**(c)**	0.0	0.0	**81.0**	2.8	7.0	7.0	0.7	1.4	0.0	0.0	0.0
lysosomes	**(d)**	0.9	0.5	3.6	**85.6**	0.0	7.7	0.9	0.5	0.0	0.5	0.0
microtubules	**(e)**	0.0	0.0	9.8	1.0	**87.3**	2.0	0.0	0.0	0.0	0.0	0.0
mitochondria	**(f)**	0.0	0.0	4.1	4.5	0.0	**90.7**	0.0	0.0	0.4	0.4	0.0
nucleoli	**(g)**	0.0	0.0	1.4	1.4	0.0	0.0	**86.5**	9.5	1.5	0.0	0.0
nucleus	**(h)**	0.7	0.0	0.0	0.0	0.0	0.7	2.7	**96.0**	0.0	0.0	0.0
peroxisomes	**(i)**	0.0	0.0	0.0	2.8	0.0	2.8	0.0	0.0	**88.7**	5.6	0.0
plasma membrane	**(j)**	2.1	0.0	0.0	1.0	0.0	2.1	0.0	0.0	0.0	**94.9**	0.0

Each row shows the classification results for a specific protein location. Therefore, a single entry denotes the fraction of images from a specific protein location (row), which were associated with a specific class label (column). In the case of a correct classification, the row index and the column index are equal. The column marked by (*) shows the fraction of protein distribution patterns which were considered as unknown.

### Retraining Task

Besides the experiments concerning a fixed set of protein locations, we analysed the ability of the proposed protein localisation technique to detect and incorporate new protein locations after training. Since feature set ℬ produced the best results with respect to the classification task, feature set A was neglected in all following experiments.

**Figure 6:**
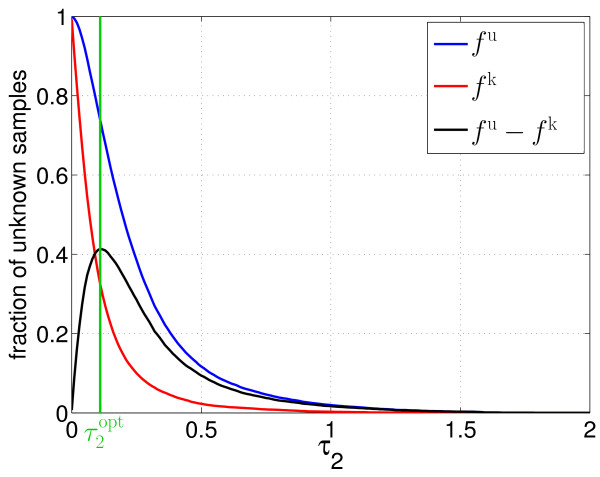
**Fractions of potentially unknown samples**. Depending on *τ*_2_, different fractions of patterns from the known and from the new cell compartments are regarded as potentially unknown. Here, the value of *τ*_2_, which maximises the difference between both fractions, is considered as optimal.

First, the fractions of patterns classified as potentially unknown were analysed (see Figure 6). Using $\tau_{2}^{\text{opt}}$, 73.9% of the unknown patterns were correctly recognised as potentially unknown (*f*^*u*^). So, a sufficient number of training samples was available for the following retraining step. On the other hand, 32.5% of the images showing known protein locations were considered as potentially unknown as well (*f*^*k*^). This means that a biological expert would have to label about one third of all images depicting known location patterns, which might be too high a number for some experiments.

In principle, alternative methods for determining an appropriate value of *τ*_2 _are possible. So, *f*^k ^could be diminished more strongly, if required. However, such a reduction entails a decline of *f*^u ^as well (cf. Figure 6). Nevertheless, modifications of *τ*_2 _might enable an adaptation of the proposed approach to a greater variety of tasks and users.

Besides the fraction of feature vectors classified as potentially unknown, the accuracy of the classifiers is critical. Here, the accuracies regarding different subsets of the considered data have to be distinguished. ${\bar{\text{ACC}}}_{\text{test}}^{\text{u}}$ denotes the mean accuracy with respect to the new protein locations after one epoch of retraining. In contrast, ${\bar{\text{ACC}}}_{\text{incorrect}}^{\text{u}}$ symbolises the fraction of images showing new location patterns that were part of the training sets and incorrectly classified as known rather than being considered as potentially unknown. Hence, these images could not contribute to the retraining. Nevertheless, a certain fraction of them is classified correctly after other feature vectors of the corresponding class have been learnt. Finally, ${\bar{\text{ACC}}}_{\text{before}}^{\text{k}}$ and ${\bar{\text{ACC}}}_{\text{after}}^{\text{k}}$ denote the mean accuracies of the known cell compartments before and after retraining, respectively.

**Figure 7:**
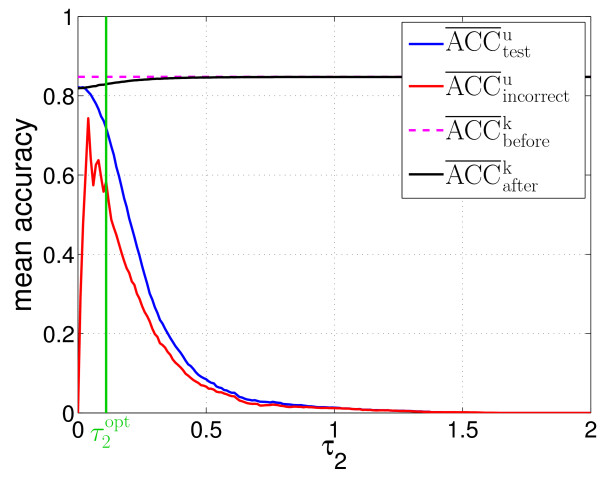
**Mean accuracies resulting from retraining the SFAM**. The mean accuracies for classifying the new cell compartments are strongly influenced by *τ*_2_. Here, the proposed threshold $\tau_{2}^{\text{opt}}$ leads to good results. The mean accuracies concerning the a priori known protein locations are only slightly affected by *τ*_2_. Moreover, $\tau_{2}^{\text{opt}}$ is considerably lower than *τ*, as *τ *usually exceeds one.

The values of ${\bar{\text{ACC}}}_{\text{test}}^{\text{u}}$ indicate that the SFAM is able to incorporate new location patterns (see Figure 7). Even difficult patterns, which closely resemble the old cell compartments (represented by 1-*f*^u^), are classified with acceptable mean accuracies ${\bar{\text{ACC}}}_{\text{incorrect}}^{\text{u}}$. Here, the choice of $\tau_{2}^{\text{opt}}$ has proven beneficial again, since it enables a good compromise between the level of classification accuracy regarding the new protein locations (${\bar{\text{ACC}}}_{\text{test}}^{\text{u}}$ = 71.9%) and the additional need for user interaction symbolised by *f*^k ^= 32.5% (cf. Figures 6 and 7). The accuracies regarding the old, known locations are barely affected by the retraining, and resemble the results known from the evaluation regarding the original classification task.

Since the number of available features has a strong impact on the classification accuracy of the SFAM, we assumed that it might affect the retraining process as well. Therefore, we arranged a reduced feature set based on the systems using a fixed set of protein locations. Such a reduced feature set was expected to reveal important information regarding the retraining process, even though it is of limited use for real applications, since no information about the unknown classes is available. Unfortunately, all results regarding the fixed set of protein locations were computed by means of five-fold cross-validation; that is, five runs using different classifiers were performed to determine mean values for the classification results. Consequently, five reduced feature sets resulted from the complete feature set. So, a single reduced feature set needed to be compiled first. Here, we exploited the fact that the reduced feature sets for a specific parameter setting are very similar, in particular, if the SDA is employed. The final features were therefore selected by a set union of the five sets yielded by the cross-validation runs. The size of these feature sets was chosen so as to maximise the mean accuracy. Due to the strong overlap of the five sets provided by the SDA, which comprise 15 features each, the resulting set encompasses only 22 features in total.

Again, the feature reduction proved beneficial. *f*^k ^decreased from 32.5% to 25.7% and the mean accuracies rose by approximately 4%, which confirms the results concerning the application of feature reduction methods known from the classification task. Only ${\bar{\text{ACC}}}_{\text{incorrect}}^{\text{u}}$ declined by about 5%, which indicates that the classification of the new protein locations is impeded in favour of the old known locations. This conclusion is, in particular, supported by the decrease of *f*^k^.

The preference of the old, known cell protein locations is caused by the training procedure: The networks were trained with the old cell compartments until their weights did not change between two subsequent epochs. So, conflicts resulting from overlapping categories belonging to different classes could be solved. Afterwards, the input vectors of the new locations were presented once. Therefore, the new locations are not as well integrated into the classifier as the old ones. This could be circumvented if samples for the known cell compartments were presented during retraining. Then, there would be no difference between batch and on-line learning. In principle, the fraction of samples measured by *f*^k ^could be employed for training the classifier to recognise the old classes. So, it constitutes no drawback if *f*^k ^does not equal zero.

### Automatic Data Generation

The influence of the procedure used for generating additional training data was measured by repeating the experiments introduced in the section concerning the classification task. But here only manually acquired protein distribution patterns were used for the training. Table 5 contrasts the resulting mean accuracies with the original results regarding the classification task (cf. Table 3). Here, the focus is on the accuracies concerning the automatically generated samples, as only these samples are biased by the cell recognition method.

**Table 5:** Localisation accuracies regarding the automatically generated patterns.

			**M**		**M+A**	
				
**feature set**	**reduction method**	**#features**	**$\bar{ACC}$**	**ACC**	**$\bar{ACC}$**	**ACC**
A	-	73	0.782	0.816	0.790	0.828
ℬ	-	73	0.794	0.836	0.806	0.835
A	SDA	17	0.867	0.883	0.873	0.887
ℬ	SDA	15	0.880	0.890	0.883	0.892

These results were obtained using only manually acquired protein distribution patterns (M) and both manually and automatically obtained patterns (M+A) for training. They correspond to column II of Table 3.

Although the impact of the automatic data generation on the mean accuracies is not significant, the results from Table 5 indicate a positive influence on the recognition of automatically determined protein distribution patterns. The total accuracies are barely affected.

## Discussion

The given results demonstrate that our approach is able to classify protein distribution patterns with a sufficient accuracy. The distribution patterns may either be obtained manually or yielded by a cell recognition approach. Although the total accuracy seems to be slightly lower than the one of related protein localisation techniques developed by Murphy's group which reach values up to 95.3% (see [3]), it must be taken into account that the proposed techniques neither rely on single-cell images nor analyse multi-cell images as a whole. Rather it is possible to analyse clustered cells individually. So, if one micrograph contains only three cells and the majority vote is chosen as the correct protein localisation, the total accuracy of the system using feature set ℬ and feature reduction by SDA would amount to 96.7%. As typical images contain more than ten cells, accuracies higher than 99.7% may be reached easily.

Furthermore, here the optimisation of relevant parameters is performed based on a different measure – the mean accuracy $\bar{\text{ACC}}$ – which, in contrast to well-known approaches, fosters an equally accurate recognition of all protein locations. But it may lead to a small decrease of the classification accuracy, since the additional constraint of similar location-specific accuracies is imposed on the classifier.

Besides analysing the classification of protein distribution patterns in a set of fixed locations, we have demonstrated that using our approach an incremental learning of new protein locations can be performed: At first, patterns from these new locations are detected as unknown and sorted out for future inspection. After the biological experiment has finished, an expert is able to examine these patterns and associate them with appropriate locations. Then, they can be incorporated into our system and used in further experiments. Even if a protein distribution pattern is sorted out, the classifier can provide information about its similarity to the known locations, which might support potential users. As an alternative to the proposed semi-automated procedure, a clustering of the unknown inputs as suggested by Murphy's group [2] could be performed. Since our classifier is based on a fuzzy ART [50] clusterer itself, clustering information could be exploited directly. But the resulting mixture of user-annotated and automatically generated classes would lead to classifiers whose results are very difficult to interpret.

## Conclusion

We have introduced a technique, which unifies several approaches that are usually investigated separately: Besides performing a feature-based protein localisation, it is adapted to a cell recognition method and enables the detection and incorporation of new protein locations, if required. Although almost the complete system can be applied in a fully-automated way, a semi-automated approach is preferred regarding the retraining with new locations in order to ensure the biological relevance of created classes. But even then, no user interaction is required during the actual analysis.

Our approach can be considered as being incremental due to two different facts: Firstly, an increasing number of cells that are visible in a fluorescence microscope image increases the accuracy of the protein localisation. Secondly, additional locations can be incorporated into the pre-trained system. Both aspects constitute an innovative contribution to the ongoing research into the subcellular localisation of proteins in living cells.

The proposed technique does not necessitate demanding microscopy or image-enhancing methods such as digital deconvolution, which facilitates its application in high-throughput experiments. Furthermore, since no fluorescence channels are employed for cell recognition or to acquire auxiliary images, they are free for co-localisation experiments that could refine the set of recognisable protein locations in close analogy to manual protein localisation procedures [5].

## Data availability

The microscope images as well as the 1,326 cell masks that were used for evaluating the proposed protein localisation technique have been summarised to the *2D Sf9 Dataset*, which is available from .

## Authors' contributions

MT drafted the manuscript and played the major role in developing the proposed protein localisation approach. NJ conducted the underlying biological experiments, provided the required microscope images and cell masks, and was involved in writing the manuscript. FK conceived of the study and edited the manuscript.
